# Use of Photobiomodulation Combined with Fibrin Sealant and Bone Substitute Improving the Bone Repair of Critical Defects

**DOI:** 10.3390/polym14194170

**Published:** 2022-10-04

**Authors:** Karina Torres Pomini, Daniela Vieira Buchaim, Ana Carolina Cestari Bighetti, Jesus Carlos Andreo, Marcelie Priscila de Oliveira Rosso, José Stalin Bayas Escudero, Bruna Botteon Della Coletta, Murilo Priori Alcalde, Marco Antonio Hungaro Duarte, Dimitrius Leonardo Pitol, João Paulo Mardegan Issa, Edilson Ervolino, Matheus Bento Medeiros Moscatel, Márcia Zilioli Bellini, Alexandre Teixeira de Souza, Wendel Cleber Soares, Rogerio Leone Buchaim

**Affiliations:** 1 Department of Biological Sciences, Bauru School of Dentistry (FOB/USP), University of São Paulo, Bauru 17012-901, Brazil; 2 Postgraduate Program in Structural and Functional Interactions in Rehabilitation, Postgraduate Department, University of Marilia (UNIMAR), Marilia 17525-902, Brazil; 3 Teaching and Research Coordination of the Medical School, University Center of Adamantina (UNIFAI), Adamantina 17800-000, Brazil; 4 Department of Dentistry, Endodontics and Dental Materials, Bauru School of Dentistry, University of São Paulo (FOB/USP), Bauru 17012-901, Brazil; 5 Department of Basic and Oral Biology, School of Dentistry of Ribeirão Preto, University of São Paulo (FORP/USP), Ribeirão Preto 14040-904, Brazil; 6 Department of Basic Sciences, School of Dentistry, São Paulo State University (UNESP), Araçatuba 16066-840, Brazil; 7 Dentistry School, University of Marilia (UNIMAR), Marilia 17525-160, Brazil; 8 Pro-Rectory of Research and Graduate Studies, University Center of Adamantina (UNIFAI), Adamantina 17800-000, Brazil; 9 Rector/President, University Center of Adamantina (UNIFAI), Adamantina 17800-000, Brazil; 10 Vice-Rector/President, University Center of Adamantina (UNIFAI), Adamantina 17800-000, Brazil; 11 Graduate Program in Anatomy of Domestic and Wild Animals, Faculty of Veterinary Medicine and Animal Science, University of São Paulo (FMVZ/USP), São Paulo 05508-270, Brazil

**Keywords:** bone repair, fibrin sealant, biomaterial, photobiomodulation therapy, low-level laser therapy, polymers, bone substitute, scaffolds

## Abstract

In this preclinical protocol, an adjunct method is used in an attempt to overcome the limitations of conventional therapeutic approaches applied to bone repair of large bone defects filled with scaffolds. Thus, we evaluate the effects of photobiomodulation therapy (PBMT) on the bone repair process on defects filled with demineralized bovine bone (B) and fibrin sealant (T). The groups were BC (blood clot), BT (B + T), BCP (BC + PBMT), and BTP (B + T + PBMT). Microtomographically, BC and BCP presented a hypodense cavity with hyperdense regions adjacent to the border of the wound, with a slight increase at 42 days. BT and BTP presented discrete hyperdensing areas at the border and around the B particles. Quantitatively, BCP and BTP (16.96 ± 4.38; 17.37 ± 4.38) showed higher mean bone density volume in relation to BC and BT (14.42 ± 3.66; 13.44 ± 3.88). Histologically, BC and BCP presented deposition of immature bone at the periphery and at 42 days new bone tissue became lamellar with organized total collagen fibers. BT and BTP showed inflammatory infiltrate along the particles, but at 42 days, it was resolved, mainly in BTP. In the birefringence analysis, BT and BTP, the percentage of red birefringence increased (9.14% to 20.98% and 7.21% to 27.57%, respectively), but green birefringence was similar in relation to 14 days (3.3% to 3.5% and 3.5% to 4.2%, respectively). The number of osteocytes in the neoformed bone matrix proportionally reduced in all evaluated groups. Immunostaining of bone morphogenetic protein (BMP—2/4), osteocalcin (OCN), and vascular endothelial growth factor (VEGF) were higher in BCP and BTP when compared to the BC and BT groups (*p* < 0.05). An increased number of TRAP positive cells (tartrate resistant acid phosphatase) was observed in BT and BTP. We conclude that PBMT positively influenced the repair of bone defects filled with B and T.

## 1. Introduction

Bone tissue presents good regenerative capacity, and, on several occasions, it has the property of restoring its original bone structure both in architecture and in physiology [1,2]. However, certain physiological, pathological, or accidental conditions associated with extrinsic factors such as smoking and chronic alcoholism can affect bone homeostasis and affect the prognosis of bone tissue regeneration [3,4,5,6].

In addition, in cases of large bone defects in which the regenerative physiological capacities are exceeded, reconstructive surgery procedures with bone grafting as an important treatment technique are required [7,8]. Among all grafts available for clinical application, autogenous bone is still considered a “gold standard”, since all the characteristics required for bone regeneration in terms of osteoinduction, osteoconduction, and osteogenesis are combined. However, the autograft presents some limitations such as limited availability and complications at the donor site [9,10].

Given these limitations, there is an increase in the search for mechanically and biologically adequate bone substitutes capable of repairing critical-size bone defects by a new tissue with morphofunctional characteristics similar to the original [11,12]. Within the available options, bovine xenografts are the second most frequently used option with promising results [13,14] because of their crystallinity, chemical composition similar to human bone, and naturally porous architecture [15].

Often, bone substitutes are incorporated into three-dimensional scaffolds, such as fibrin sealants, to form a graft mixture moldable to the receptor bed, preventing the dispersion of particles and providing structural stability, favoring the adhesion of bioactive molecules and cells, and providing conditions for proliferation and cell differentiation [16]. Among the commercially available fibrin sealants, human blood derivatives have been used in experimental and clinical studies, composed of fibrinogen and human factor XIII, reconstituted in antifibrinolytic solution (aprotinin), in addition to human thrombin reconstituted in calcium chloride solution [17,18].

In recent years, innovative modalities, such as low-intensity pulsed ultrasound, photobiomodulation therapy (PBMT) with low-level laser, and hyperbaric oxygen therapy, or their combination, are being evaluated in an attempt to improve metabolism and accelerate the bone repair process [19,20,21,22]. Photobiomodulation has been shown to be capable of biostimulation of bone cells during in vitro or in vivo studies [23,24,25]. The most accepted hypothesis in relation to the mechanism of action is that monochromatic laser photons activate chromophores in the cell’s cytochrome C oxidase, resulting in accelerated cellular activity, increased ATP and alkaline phosphatase (ALP) concentrations, and calcium release [25,26].

Despite the previous existence of publications on the use of three-dimensional scaffolds in regenerative medicine and the growing interest in adjuvant methods that seek to overcome the limitations of conventional therapeutic approaches for bone repair, few studies have investigated the effect of PBMT associated with biomaterials on the process of bone regeneration.

Therefore, we aim to evaluate the effects of PBMT in the repair of critical bone defects in rat calvaria filled with bone substitute and fibrin sealant, by means of qualitative and quantitative microtomographic, histomorphological studies using Masson’s trichrome and Picrosirius red staining, and immunohistochemical analysis with BMP—2/4, OCN, VEGF and TRAP immunomarkers.

## 2. Materials and Methods

### 2.1. Bone Graft Substitutes—Xenograft

We used the inorganic matrix Bio-Oss™ (Geistlich Pharma AG, Wolhusen, Switzerland), from bovine bone cortical material with a calcium-phosphorus ratio and a structure very close to human hydroxyapatite (Ministry of Health Registration ANVISA No. 806.969.30002), with the following physicochemical characteristics: granules of cancellous bone (0.25–1 mm); demineralized bovine bone (B) matrix–xenograft; sterilization, γ-irradiation; pores sizes, macropores (300–1500 µm) and micropores (size of Haversian and vascular marrow canals); amount, 2.0 g vial; purification process, multi-phase (thermal deproteinization processes, temp < 900); intracrystalline spaces, 3–26 nm; porosity, 70–75% of the total volume; internal surface area, 100 m^2^/g; and compressive strength, 35 MPa.

### 2.2. Fibrin Sealant—Human-Blood Derived

The fibrin sealant Tisseel (T) has part of its constituents derived from human plasma. Tisseel Lyo™ is the trademark manufactured by Baxter Healthcare Ltd., Norfolk, United Kingdom, with Ministry of Health Registration ANVISA No. 1.0683.0182. It is available in four glass vials containing: component 1 containing Tisseel Lyo powder (sealant protein)—freeze-dried, with 91 mg/mL human fibrinogen; component 2 containing powdered thrombin—freeze-dried, with 500 IU/mL human thrombin; component 3 containing aprotinin—diluent solution of component 1 containing 3000 UIC/mL synthetic aprotinin; component 4 containing calcium chloride solution—diluent for component 2 containing 40 μmol/mL of calcium chloride. Tisseel Lyo™ contains 0.6–5 IU/mL human coagulation factor XIII, which is isolated from plasma together with human fibrinogen.

**Figure 1:**
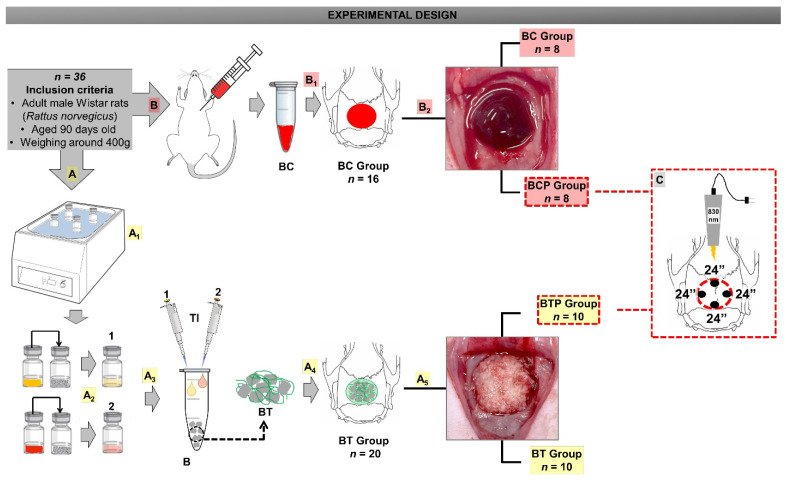
Allocation of the specimens in two large groups: BC and BT. (**A**) Preparation and reconstitution of Tisseel^TM^. (**A_1_**) The fibrin components vials were preheated. (**A_2_**) Mixture of fibrin sealant components: sealant protein concentrate plus aprotinin solution (1) and freeze-dried human thrombin plus calcium chloride solution (2). (**A_3_**) B was mixed with the reconstituted solutions (1 and 2). (**A_4_**) BT group (B plus T). (**A_5_**) Subdivision: BT (without PBMT) and BTP (with PBMT). (**B**) Blood collection by cardiac puncture. (**B_1_**) BC group—the defects were filled with blood from cardiac puncture. (**B_2_**) Subdivision: BC (without PBMT) and BCP (with PBMT). (**C**) Illustrative figure: 90° laser emitter, 4-point application, time 24 s/point.

In preparation and reconstitution of the fibrin sealant, all vials were initially preheated in a water bath for approximately 3 min at a temperature of 33–37 °C, controlled by a mercury thermometer (Thermometers Labor™, São Paulo, Brazil). The aprotinin solution was then transferred to the flask containing the freeze-dried Tisseel Lyo™ powder and the calcium chloride solution into the vial containing freeze-dried thrombin, shaking rapidly, but avoiding foaming. The flasks with the solutions of sealant protein and thrombin solution were then returned to the water bath for one minute to be used. The solutions were maintained at this temperature throughout the surgical procedure until the moment of use within 4 h after reconstitution (Figure 1A).

### 2.3. Experimental Design

Thirty-six Wistar rats were obtained from the bioterium of the Ribeirão Preto (University of São Paulo—USP, Brazil), following the inclusion criteria: Wistar rats (*Rattus norvegicus*), adult male, healthy, and weighing around 400 g.

The animals were received at 42 days of age and, during the entire experimental period, they were housed in cages containing four animals each (Bioterium of Bauru School of Dentistry—USP, Brazil). The cages had feeders and drinkers “ad libitum” (Nuvilab^TM^ rat chow, Nuvital, Colombo, Brazil and filtered water) in an air-conditioned environment, period dark–light 12/12 h, humidity 60% ± 10, air exhaustion, lighting 150 lux/1 m floor, maximum noise 70 dB (decibel), and temperature 22 °C ± 2 °C.

This study was carried out with approval of the Institutional Review Board on Animal Studies of Bauru School of Dentistry, USP, Brazil (Protocol No. CEEPA-019/2016; 3 October 2016). This preclinical study was performed according to the ARRIVE (Animal Research: Report of in vivo Experiments) guidelines [27] and experimentally designed on the principles of NC3Rs (National Center for Replacement, Refinement, and Reduction of Research Animals). During all experimental phases, the animals were monitored for apathy, pain expression, aggressiveness and overexcitement, symptoms of depression, and other characteristics that may have altered their usual behavior. Possible changes in gait, facial expression and posture were also investigated. It was also observed if the animals had any unusual behaviors such as changes in water and food consumption, as well as possible clinical symptoms.

The 90-day-old animals were randomized into two broad groups: BC, *n* = 16 (blood clot, surgical cavity with blood clot) and BT, *n* = 20 (surgical cavity with demineralized bovine bone and fibrin sealant) and that these groups were further subdivided into BC, *n* = 8 (surgical cavity with blood clot without PBMT), BCP, *n* = 8 (surgical cavity with blood clot and PBMT), BT, *n* = 10 (surgical cavity with demineralized bovine bone and fibrin sealant without PBMT), and BTP, *n* = 10 (surgical cavity with demineralized bovine bone and fibrin sealant and PBMT), according to the treatment provided (Figure 1).

### 2.4. Surgical Procedures

The same team of professionals performed all surgical procedures. The surgeries were performed under general anesthesia with Xylazine 10 mg/kg (Anasedan™, Ceva, Paulínia, Brazil) and Ketamine 50 mg/kg (Dopalen™, Ceva, Paulínia, Brazil). A semi-lunar incision was made to expose the cortical bone extensively and thus to avoid damages to the periosteum and adjacent tissues by the trephine.

Craniotomy was then performed using an 8 mm diameter trephine drill (Neodent™, Curitiba, Brazil) with continuous irrigation, sterile saline solution 0.9% sodium chloride JP^TM^ (JP Farma—Pharmaceutical Industry, Ribeirão Preto, Brazil) to avoid osteonecrosis, in the center of parietal bones, including the sagittal suture [28].

The surgical cavities in the BC group received 0.25 mm^3^ of cardiac puncture blood (Figure 1B_1_) [29]. In BT, they received 0.1 mm^3^ (equivalent to 0.03 mg) of B incorporated into 40 µL of fibrinogen solution reconstituted in aprotinin and 40 µL of thrombin solution reconstituted (Figure 1A_4_). The amounts of demineralized bovine bone and fibrin sealant used were previously established in a pilot study.

After the grafts had been repositioned, the periosteum and overlying tissues were repositioned to reduce the risk of invagination into the bone defect [30].

For post-surgical recovery, the animals were exposed to incandescent light and received a single oral dose of paracetamol 200 mg/kg (Paracetamol, Medley, São Paulo, Brazil) dissolved in water available in the cages.

### 2.5. Photobiomodulation Therapy Protocol

Photobiomodulation with a 830 nm gallium–aluminum–arsenide (GaAlAs) laser (Laserpulse^TM^ IBRAMED, Amparo, Brazil) (Figure 1C) was used to apply low-level laser therapy (LLLT) transcutaneously, contact and static mode at four point with the hand piece positioned perpendicular to the surgical area. The animals of Groups BCP and BTP underwent laser irradiation with the following parameters (Figure S1, Supplementary Materials) [21,28].

### 2.6. Euthanasia

The animals were randomly divided to be euthanized after 14 and 42 days of the surgical procedure. They received an excessive dose of xylazine/ketamine, following the guidelines of the National Council for the Control of Animal Experimentation (CONCEA/Brazil). In the BC and BCP groups, 4 animals/period; in BT and BTP, 5 animals/period. The skin-coated skulls were collected and fixed in 10% phosphate-buffered formalin (Allkimia^TM^—Commerce of Materials for Laboratories LTDA, Campinas, Brazil) for 48 h and subsequently sent for microtomographic examination.

### 2.7. Micro-CT Scanning

After fixation in formalin solution, the specimens were subjected to X-ray beam scan in the computed microtomographic machine SkyScan 1174v2 (μ-CT, Bruker micro-CT^TM^, Kontich, Belgium). The images were captured with 0.73° at each pace, 13.76 µm voxel, and further reconstructed using NRecon^TM^ v.1.6.8.0, SkyScan, 2011 (Bruker micro-CT^TM^, Kontich, Belgium). Ring artefact and beam hardening corrections were applied in reconstruction. Afterward, the reconstructed images were realigned using DataViewer^TM^ 1.4.4.0 software (Bruker micro-CT^TM^, Kontich, Belgium).

The region of interest was chosen as a sleeve around new-formed bone (NFB) and bone particles individually with 0.5 mm of thickness in the plan coronal, using the CTAn^TM^ 1.10.1.0 software (SkyScan, Kontich, Belgium), based on the average diameter and minimum height (8 × 0.8 mm) obtained between the samples, to quantify the structures. Subsequently, the binarization of the images (separation of the biomaterial and NFB phases) was performed with the upper and lower limits (grayscale threshold) for neoformed bone 130–72 and biomaterial 130–255, and then the volume of each phase. Afterward, the bone volume (BV), percent bone volume (bone volume/trabecular volume, BV/TV), and bone mineral density (BMD) were measured for each dataset and the remaining particle percentages were taken by subtracting the bone volume (BV/TV) of the bigger amount of bone particles from the bone volume (BV/TV) of total amount of bone particles (Figure S2, Supplementary Materials).

### 2.8. Histotechnical Processing

Subsequently, the pieces were washed in tap water for 24 h and placed in 10% ethylenediaminetetraacetic acid (EDTA) solution containing 4.13% Titriplex^TM^ III (Merck KGaA, Darmstadt, Germany) and 0.44% sodium hydroxide (Labsynth^TM^, São Paulo, Brazil) for a period of 7 weeks. Specimens were submitted to standard histological procedures for embedding in Histosec^TM^ (Merck KGaA, Darmstadt, Germany).

Semi-serial sections with a 5 μm thick coronal plane were used to reach the center of the 8 mm defect using the scale free size tool available in DP controller software (3.2.1.276—2001-2006, Olympus Corporation^TM^, Tokyo, Japan). Subsequently, the sections were stained by Masson’s trichrome, hematoxylin, and eosin (for analysis of osteocytes), picrosirius red solution and against Harris hematoxylin stain (for evaluation of total collagen fibers, type 1 and 2), and immunostaining for markers of bone formation and resorption (vascular endothelial growth factor, VEGF; bone morphogenetic protein; BMP—2/4; osteocalcin, OCN; tartrate-resistant acid phosphatase, TRAP).

### 2.9. Histological Evaluation of Defects Bone Healing

The histologic sections were analyzed by light microscopy (Olympus^TM^ model BX50) at approximate magnifications of 10 and 40×. In sections stained with Masson’s trichrome, the evaluation consisted of description of the healing events such as granulation tissue, bone neoformation and remodeling, inflammation, and the interaction between the biomaterial bone graft and newly formed bone.

This staining makes it possible to differentiate original mature bone, stained red, and newly formed (young) bone, which stains green. This technique is also used for connective tissue, staining its collagen fibers in green, highlighting them from other tissue elements.

### 2.10. Birefringence Analysis of Collagen Content of Bone Healing Defects

Picrosirius red staining was used to determine the quality and quantity of a new organic matrix in the studied periods of 14 and 42 days of repair. Images were obtained from the defect using higher-resolution Leica DFC 310FX camera (Leica^TM^, Microsystems, Wetzlar, Germany) connected to confocal laser microscope and capture system (Leica DM IRBE, Leica^TM^, Microsystems, Heerbrugg, Switzerland). Three histological fields (10× magnification) were captured corresponding fully to the defect. All remaining bone present in the histological fields and removed from the images were then selected to avoid counting these fibers in Adobe Photoshop CS6 software.

The Images were transferred to AxioVision Rel. 4.8 Ink analysis software (Carl Zeiss MicroImaging^TM^ GmbH, Jena, Germany) and the total area, the biomaterial area of the particles and the area of connective tissue plus newly formed bone, was determined with dashed lines, yielding values in pixels^2^. The birefringence analysis of the collagen content of bone healing defects is shown in Figure S3 (Supplementary Materials).

Using the interactive processing–segmentation–threshold tool, the RGB color standard was determined for each color. Then, the density area or percentage (%) area analysis of each fiber type per color was assessed. Woven bone was recognized by its random, unorganized fibrillar pattern, usually with polarization colors ranging from red-orange (poorly organized woven bone) and lamellar bone (bright green/yellow), depending on fiber width.

### 2.11. Quantitative Analysis of Osteocytes in Newly Formed Bone Tissue

The analysis of osteocytes was performed in two pre-established areas referring to the new bone tissue formed at the edges of the lesion site. The hematoxylin- and eosin-stained images were captured at 20× magnification and evaluated using the AxioVision Rel. 4.8 Ink software (Carl Zeiss^TM^ MicroImaging GmbH, Jena, Deutschland). The areas of bone neoformation and medullary channels were manually surrounded with dashed lines to determine the total matrix area in pixels^2^ (px^2^ = 0.528 mm^2^). Then, three horizontal lines were drawn from each total matrix area to count the osteocyte nuclei in each one. The collected data were analyzed using Microsoft Excel 2016 and GraphPad Prism^TM^ Software, Inc, with significance level set at *p* < 0.05 (Figure S4, Supplementary Materials).

### 2.12. Immunohistochemical Processing

Standard deparaffinization, rehydration, and antigen-recovery procedures were used. Briefly, xylene and ethanol were used to deparaffinize and rehydrate the tissues and, for antigen recovery, the tissues were incubated in a sodium citrate buffer in a pressurized chamber (Decloaking chamber, Biocare Medical^TM^, Concord, CA, USA) at 95 °C for 20 min. Then, endogenous peroxidase and nonspecific sites were blocked using 3% hydrogen peroxide for 1 h and bovine serum albumin (Sigma Aldrich^TM^ Chemical Co, St. Louis, MO, USA) for 12 h, respectively.

Histological sections were divided into four batches and each batch was incubated with one of the following primary antibodies: anti-VEGF (dil. 1:200, sc-7269, Santa Cruz Biotechnology^TM^, Dallas, TX, USA), anti-BMP 2/4 (dil. 1:200, sc-9003, Santa Cruz Biotechnology^TM^, Dallas, TX, USA), and anti-OCN (dil. 1:100, sc-365797, Santa Cruz Biotechnology^TM^, Dallas, TX, USA) e anti-TRAP (dil. 1:200, sc-376875, Santa Cruz Biotechnology^TM^, Dallas, TX, USA). For signal amplification, the sections were incubated with biotinylated secondary anti-mouse/rabbit IgG antibody generated for 2 h (BA-1400, Vector Laboratories^TM^, Burlingame, CA, USA) and subsequently treated with streptavidin conjugated to horseradish peroxidase—HRP (SA-5004, Vector Laboratories^TM^, Burlingame, CA, USA) for 1 h. The development was carried out using the 3,3′-diaminobenzidine tetrachlorhydrate (SK-4105, Vector Laboratories^TM^, Burlingame, CA, USA) as a chromogen. Counterstaining with Harris hematoxylin was performed for the samples used to detect VEGF, BMP—2/4, and OCN, and counterstaining did not use counterstaining in the samples used to detect TRAP, to assist in the counting of immunostained cells. As negative control, the specimens were submitted to the same procedures, eliminating the use of the primary antibody.

This evaluation was executed with the aid of an optical microscope (Axio Scope^TM^, Carl Zeiss Microscopy GmbH, Göttingen, Germany) with attached digital camera (AxioCam^TM^ MRc5, Carl Zeiss Microscopy GmbH, Dublin, CA, USA), connected to the microcomputer, at 40× and 100× magnification of the original augmentation, over the entire length of the bone defect.

The images of the appropriate histological slides were captured using the software ZEN 2 (Blue edition; version 6.1.7601; Carl Zeiss Microscopy GmbH, Dublin, CA, USA). The extent of staining was recorded by a trained and blinded examiner for the experimental groups. For VEGF, BMP—2/4 and OCN, a semi-quantitative analysis was performed using three histological sections of each animal.

The scores for quantification were as follows [31,32]: score 0 = absence of immunostaining; score 1 = low immunostaining (less than 1/3 of the immunoreactive cells and weak marking in the extracellular matrix); score 2 = moderate immunostaining (about half of the immunoreactive cells and moderate staining in the extracellular matrix); and score 3 = high immunostaining (more than 2/3 of the immunoreactive cells and strong marking in the extracellular matrix). For TRAP, a quantitative analysis was performed using three histological sections of each animal in the original 400× magnification. TRAP-positive cells were expressed per mm².

### 2.13. Statistical Analysis

All tests were performed using GraphPad Prism version 5.00 for Windows (GraphPad Software^TM^, San Diego, CA, USA). First, the data obtained were subjected to a normality test (Kolmogorov–Smirnov) and homogeneity of variance (Bartlett’s test). The data homogeneously distributed (percentage of bone, materials and connective tissue, osteocyte number, and TRAP + cell number) were subsequently subjected to parametric analysis: one-way ANOVA followed by the Tukey test to compare different treatments (BC vs. BCP vs. BT vs. BTP) for each experimental period, and a “t” test to compare the influence of time (14 or 42 days) for each treatment.

The percentage data obtained for the micro-CT and histology were submitted to Pearson’s correlation test. For nonparametric data (BMP, VEGF, and OCN scores), Kruskal–Wallis followed by Dunn’s post-test and the Mann–Whitney test were used because of the non-normal distribution of the data. The tests were performed with a 5% statistical significance level (*p* < 0.05).

## 3. Results

Regarding the animals used in this preclinical experiment, there were no complications that needed to be reported, as well as no disease or evident sign that led to the removal of an animal (clinical outcome).

### 3.1. µ-CT Analysis at 14 and 42 days

**Figure 2:**
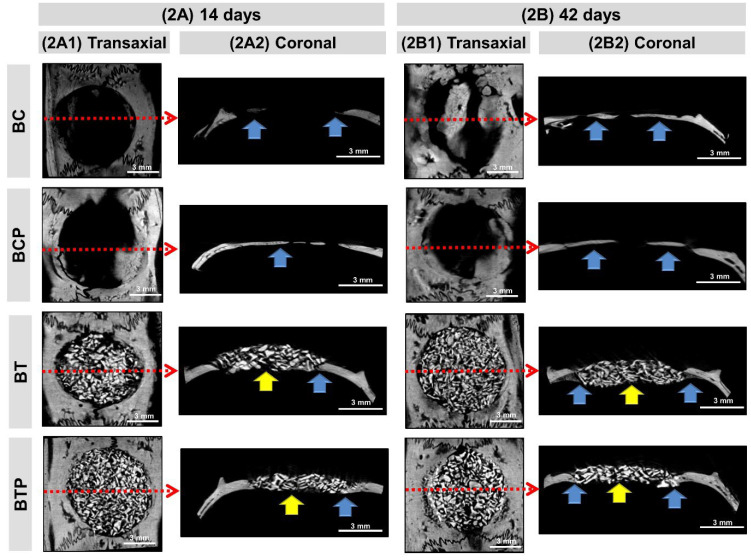
Healing of rat calvarial defects assessed by micro-CT. (**2A**,**2B**) 14 and 42 days: 2D views of transaxial (**2A1**–**2B1**), coronal (**2A2**–**2B2**) sections. Images show the evolution of the repair of defects filled with clot (BC and BCP) and fibrin sealant plus demineralized bovine bone (BT and BTP). Biomaterial particles (yellow arrow), newly formed bone tissue at the defect edges and under the dura mater (blue arrow) and red-dotted arrow (transaxial) indicate the central region of the defect corresponding to the coronal section. All scaled image sized 3 mm.

The descriptive analysis of the microtomographic images is shown in Figure 2 (transaxial, 2A1–2B1, and coronal, 2A2–2B2 sections). In all groups, as observed in the coronal and transaxial sections, they presented a pattern of bone growth from the edges of the irregular defect in their distribution.

In the 14-day period, a hypodense cavity was observed in both the BC and BCP groups, which corresponded to the bone fragment removed, laterally delimited by the hyperdense bone border and by hyperdense regions related to the centripetal growth of the newly formed bone tissue. In the groups BT and BTP, every area of the defect was filled by hyperdense xenograft particles and correctly positioned, without extravasation.

At 42 days, evaluating all extension of the defect in the BC and BCP groups, an increase was observed in the hyperdensity of the area near the edges of the defect and some islets, with a more organized structure mainly in the BCP group. In the same period, the BT and BTP groups presented the defects still completely filled with the xenograft particles, with a discrete appearance of fine bone trabeculae on the edge of the defect and overlapping the dura mater.

The morphometric data of the 3D microtomographic images of volume and percentage obtained in the CTAn program (Skyscan^TM^ Company, Kontich, Belgium) are presented in Table 1. The defect/graft volume, total volume (TV), of the clot-filled groups (BC and BCP) was related to the volume of the bone block surgically removed, while the TV of the groups filled with biomaterial (BT and BTP) was of the grafted volume. Thus, at 14 days, TV in BT and BTP (mean 113.41 mm^3^) was 212% greater than that of the defect in groups BC and BCP (mean 36.37 mm^3^) and did not change at 42 days.

Regarding the material volume (MV), only in the BT and BTP groups the biomaterial particles (MV of 40 mm^3^) occupied 35.89% of the TV, with no differences between groups and periods.

In the analysis of bone volume (BV) in all experimental groups, bone formation was discreet and occurred near the edge of the defect and on the surface of the dura mater. In the groups filled with clot (BC and BCP), at 14 days the BV/TV averaged 8.11% (BV = 2.98 mm^3^) and increased to 15.69% (BV = 5.68 mm^3^) at 42 days. Meanwhile, in the groups treated with biomaterial, they were similar between groups and periods, occupying 15.97% (BV = 17.84 mm^3^).

Hypodense images (black) referred to connective tissue, StV, as seen in histological sections. At 14 days, in the BC and BCP groups, the soft tissue occupied 91.89% (StV = 33.4 mm^3^) of the defect, decreasing to 84.51% (StV = 30.25 mm^3^) at the end of the experiment because of the increase in bone formation. In the BT and BTP groups, due to the presence of biomaterial particles, the StV/TV was only 48.13% (StV = 54.65 mm^3^), with no differences between groups and periods.

### 3.2. Histological Evaluation of Defects Bone Healing

**Figure 3:**
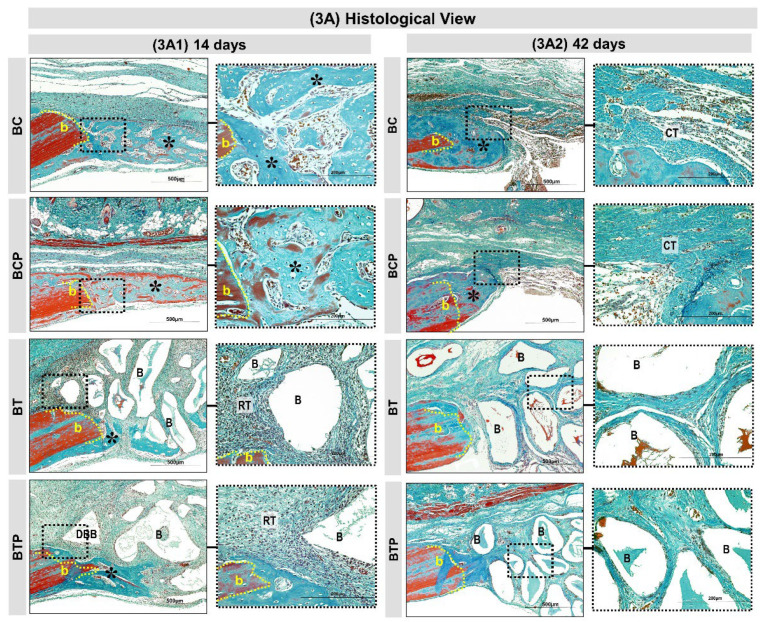
Histological views (**3A**) in calvaria defects created in the animals. BC and BCP treated with blood clot and BT and BTP treated with demineralized bovine bone plus fibrin sealant at experimental periods 14 and 42 days (**3A1**,**3A2**). At 14 days: BC and BCP groups showed bone growth (asterisks) adjacent to the defect border (b) and on the dura-mater surface. The BT and BTP groups showed a discrete bone growth (asterisks) on the border and the defect was filled by graft particles (demineralized bovine bone) surrounded by a reactional tissue (RT) containing some inflammatory cells (**3A1**). At 42 days: BC and BCP both groups showed similar bone formation with gradual increase in thickness of trabeculae, leading to a compact structure limited to the defect border. Closure of large part of the defect by fibrous connective tissue (CT). In BT and BTP groups, the graft particles and inflammatory process decreased but did not disappear and only small bone formation was present on the lesion border (**3A2**). (Masson’s trichrome; original magnification 10×; bar = 500 µm and Insets, magnified images 40×; bar = 100 µm).

At 14 days, the BC and BCP groups presented a thin layer of newly formed bone on the dura mater surface, with the presence of total collagen fibers parallel to the long axis of the created defect and granulation tissue rich in cells and blood vessels. The BT and BTP groups presented discrete new bone formation at the defect border and small osteoid matrix locus surrounding the demineralized bovine bone particles. Inflammatory infiltrate diffusely distributed in the interstitium was observed. The BTP group had small osteoid matrix locus, with more organized total collagen fibers (Figure 3A1).

At 42 days, the BCP group exhibited a slight increase in the thickness of new bone tissue, already with characteristics of diploid and presence of organized total collagen fibers in relation to the BC group. In both groups, no specimen presented complete closure of the defect or restoration of the original calvaria thickness (Figure 3A2).

In the same period, the BT and BTP groups presented tissue reaction in resolution phase, with few inflammatory cells. The height of the graft area was maintained throughout the experiment. The bone formation was confined to the defect edges and overlying the dura mater, but with higher bone density and denser and lamellar arrangement in the BTP group (Figure 3A2).

### 3.3. Picrosirius Red Staining Showed Less Bone Collagen Organization/Maturation

**Figure 4:**
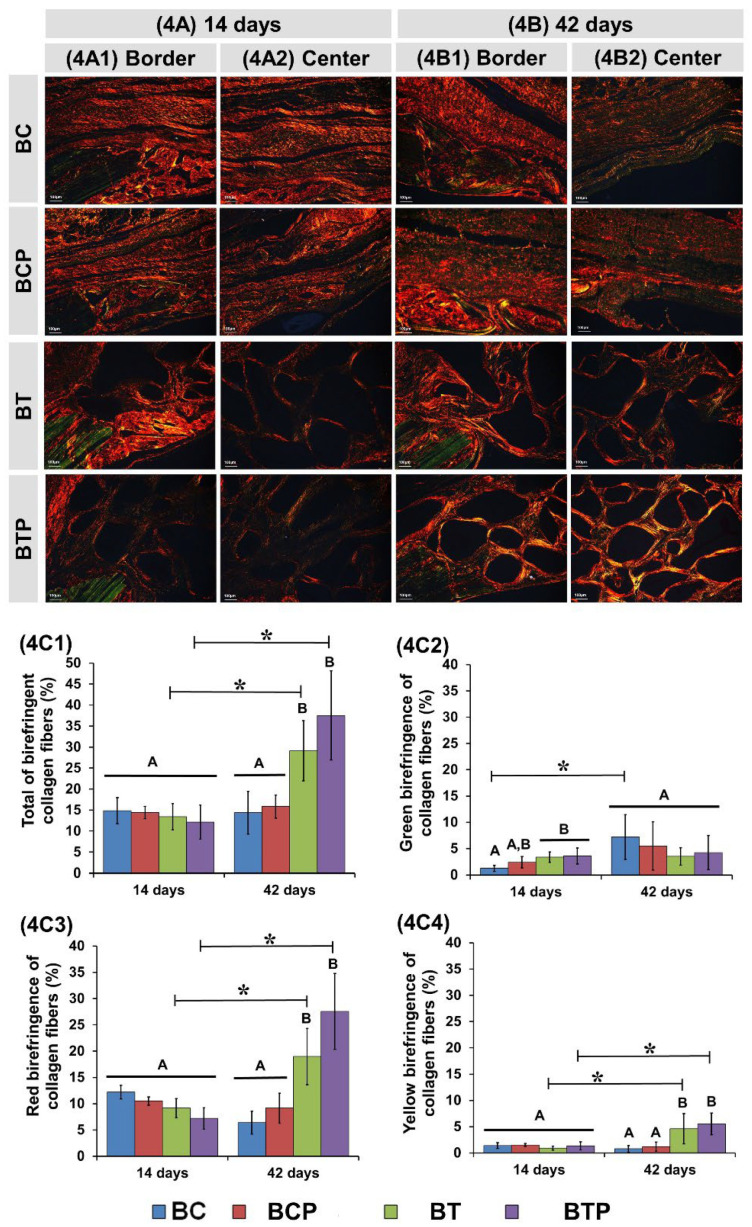
Histological images of birefringent fibers stained with picrosirius red at 14 and 42 days of repair (**4A**–**4B**). Images of the defect border and in the central region in two experimental periods (**4A1**–**4A2**, **4B1**–**4B2**). The red-orange birefringence color with immature bone formation containing thin and disorganized collagen fibers. Yellow-green color was associated with lamellar/mature bone. Original magnification 10×, scale bar 100 µm. Birefringence analysis of collagen fibers (**4C**): graphs of total of birefringence collagen fibers (**4C1**), green birefringence of collagen fibers (**4C2**), red birefringence of collagen fibers (**4C3**), and yellow birefringence of collagen fibers (**4C4**) in the bone tissue for each group. *n* = 8/group and periods (BC and BCP) and *n* = 10/group and periods (BT and BTP). * Standard deviation and different letters *p* < 0.05 between periods/group (ANOVA) and groups/period (“t” test).

At 14 days, representative images of picrosirius red staining showed similar predominance of red-orange birefringence in all experimental groups, corresponding to fine and disorganized collagen fibers with higher mean in the BC group (average 12.2%), but without significant difference in relation to the other BCP, BT, and BTP (means 10.5%, 9.14%, and 7.21%, respectively) (Figure 4A1,A2,C3).

At this period, the percentage of green birefringence fibers was higher in the BTP group (average 3.59%) compared to the other experimental groups, with significant difference from the BC group (mean 1.23%), but without statistical difference in relation to BCP and BT (2.38% and 3.37%, respectively) (Figure 4A1,A2,C2).

Regarding the percentage of fibers with yellow birefringence, there was no significant difference in all groups analyzed (Figure 4A1,A2,C4).

At 42 days, red-orange birefringence gradually decreased in the BC and BCP groups (means 12.2% to 6.4% and 10.5% to 9.16%, respectively), but without significant difference, while green birefringence increased proportionally (means 1.2% to 7.2% and 2.4% to 5.5%, respectively) (Figure 4B1,B2,C3). In the BT and BTP groups, the percentage of red birefringence increased (means 9.14% to 20.98% and 7.21% to 27.57%, respectively), but green birefringence remained similar in relation to the previous period (means 3.3% to 3.5% and 3.5% to 4.2%, respectively) (Figure 4B1,B2,C2,C3).

### 3.4. Quantitative Analysis of Osteocytes in Newly Formed Bone Tissue

Osteocyte density was obtained using the ratio of the number of osteocytes to the bone area (mm^2^). The comparative evaluation between the experimental groups, at 14 and 42 days, did not reveal a statistically significant difference (*p* = 0.9265 and *p* = 0.1609, respectively). In contrast, in evaluating the influence of time within each group, the same pattern was observed with a significant decrease in osteocyte density values in BC, BCP, BT, and BTP at 42 compared to 14 days (Table 2).

### 3.5. Immunolabelling Findings for BMP—2/4, VEGF, OCN and TRAP

The immunohistochemical technique used for the detection of VEGF, BMP—2/4, OCN, and TRAP showed high specificity in the detection of such proteins, which was proven by the total absence of labeling in the negative control. The immunoreactive cells showed a dark brown color confined exclusively to the cytoplasm, in the case of TRAP, and confined to the cytoplasm and, to a lesser extent, to the extracellular matrix, in the case of BMP—2/4, VEGF and OCN.

**Figure 5:**
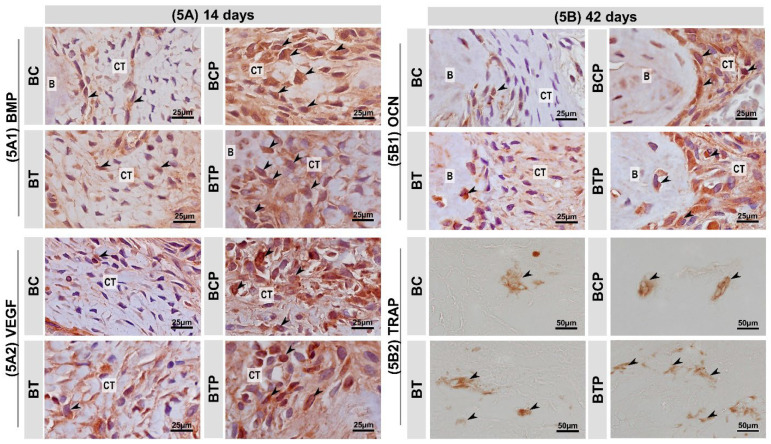
(**5A**,**5B**) Histological sections showing the appearance representative of immunolabeling after 14 and 42 days respectively in experimental groups BC and BCP treated with blood clot biostimulated by laser or not and BT and BTP treated with demineralized bovine bone plus fibrin sealant biostimulated by laser or not. The black arrows point to places where the brown spot marks the proteins: (**5A1**) immunolabeling for bone morphogenetic protein (BMP—2) in bone defects at 14 days. BMP—2/4—positive cells (arrows); connective tissue (CT). (**5A2**) Immunomarking pattern for vascular endothelial growth factor (VEGF) in bone defect at 14 days. VEGF—positive cells (arrows); connective tissue (CT). (**5B1**) Immunomarking pattern for osteocalcin (OCN) in bone defect at 42 days. OCN—positive cells (arrows); connective tissue (CT) and bone tissue (B). (**5B2**) Immunomarking pattern for tartrate-resistant acid phosphatase (TRAP) in bone defect at 42 days. TRAP—positive cells (arrows). Harris’ hematoxylin counterstaining (Scale bars: 25 μm and 50 μm; original magnification: 40× and 100×, respectively).

In BMP—2/4, during the entire study period, BMP—2 was mainly expressed in connective tissue at the margins of the defect, around the biomaterial particles and in the matrix of new bone. It presented the same immunostaining pattern at 14 and 42 days, with the BCP and BTP groups having a moderate pattern, with no statistical difference between them, in contrast to the non-biostimulated groups, with a low pattern (Figure 5A1 and Table 3).

In VEGF, positive staining for angiogenic growth factor was observed in all groups, being more pronounced in BCP and BTP in the initial phase, but with no difference between them. Thus, at 42 days, the immunostaining of these groups by VEGF ranged from moderate to high, with no statistical difference between them (Figure 5A2 and Table 3). In the OCN immunomarker, the presence of positive staining for this transcription factor in the tissue and around the biomaterial particles was evaluated in all groups. At 14 days, the biostimulated groups, BCP and BTP, showed a moderate pattern of immunostaining, but without statistical difference between them. The BC and BT groups showed a low staining pattern. At 42 days, the BTP group showed a statistically significant increase in the positive staining of the protein, close to the mineralized bone matrix, compared to the other groups (except BCP), which remained with the same pattern until the end of the experiment (Figure 5B1 and Table 3).

Regarding the immunomarker TRAP, comparatively between groups, at 14 days, it was found that the BC group had a lower number of positive TRAP cells (2.8 ± 0.84) statistically significant compared to BCP, BT, and BTP (4.8 ± 0.83; 20.2 ± 4.02 and 30.4 ± 1.34, respectively, *p* = 0001). Additionally, the same immunostaining profile was found at 42 days. Throughout the experimental period, an influence of time was noted on the increase in the number of cells with positive staining for TRAP activity only in the BTP group (30.4 ± 1.34 vs. 36.6 ± 4.51, *p* = 0.0185) (Figure 5B2 and Table 4).

## 4. Discussion

Laser photobiomodulation therapy directly interferes with tissue healing, increasing local circulation, cell proliferation, and collagen synthesis [33,34,35]. However, few studies report its influence on the bone repair process of critical defects filled with bone substitute materials associated with fibrin sealants. Thus, the methodology used in this study showed that laser photobiomodulation favored the bone repair process of critical defects filled with xenograft and derived from human blood fibrin sealant in calvaria of rats.

Animal calvaria is an anatomical region widely used in experimental models of critical-size bone defects because of morphological and embryonic similarity to the craniofacial region [36,37,38]. It is an area with limited mechanical forces and relative stability of adjacent structures, providing a favorable microenvironment for analysis of the interaction between biomaterials, newly formed bone, and the remaining bone [39].

In the search to aid the repair of defects, GaAIAs laser photobiomodulation therapy has been indicated as an adjunct to the surgical technique because it is able to reach deeper tissues because of malabsorption by water and skin pigments [40,41]. In this study, the therapy started after the surgical procedure because it is believed that a laser exerts better results in the initial phase of repair, since research in the area has a preference for postoperative transcutaneous irradiation for increasing angiogenesis, gene expression, and proteins intrinsically related to the bone repair process [42,43]. Other studies further claim that a laser improves the quality and quantity of the bone formed at this stage [44].

The infrared wavelength (830 nm) in continuous mode was chosen because of its lower loss, which can reach up to 37% of its intensity after a depth of 2 mm [45]. With the knowledge that the pericranial soft tissues that cover the parietal bones present thin thickness, it is believed that the dispersion of photons is minimal. The decision to use this laser therapy protocol is based on previous studies with satisfactory results [21,28,46,47]. However, there is no consensus as to the ideal protocol to be applied in an experimental model of defect in rat calvaria, which may conflict with the results found in different studies that were due to the difficulty of standardizing the methods.

In a preliminary study by our group of researchers, previously published, with qualitative microtomography (2D), histomorphological, and histomorphometric evaluations of new bone tissue on slides stained with hematoxylin and eosin (HE), it was possible to conclude that the support system (biocomplex), formed by fibrin sealant and xenograft, associated with the proposed PBMT protocol, created a positive microenvironment in the bone repair process [28].

In addition, the scientific literature reports numerous intrinsic characteristics of these materials directly correlated with the mechanism of action in the bone regeneration process, suggesting, when associated, the formation of a tissue construction with synergistic effects [16,47,48,49,50,51,52,53,54,55] (Table 5).

From this, we decided to deepen with the realization of this new study with new analyses, to corroborate or not these findings through new evaluations. 3D microtomographic quantification was performed to provide a detailed view of bone growth from end to end of the defect [56]. A new histological evaluation was also performed using Masson’s trichrome stain to accurately differentiate the original mature bone tissue (red) from newly formed immature bone (green) [57,58]. Qualitative and quantitative analysis of the birefringence of collagen fibers was also performed to assess the stage of maturation of the fibers present in the bone tissue by means of polarized microscopy [59,60]. In addition, quantification of osteocytes, immunostaining with BMP—2/4, VEGF, OCN, and TRAP, qualitative and quantitative, were also evaluated. To enable the comparative effect of the results, we used the same principles of the preliminary study, with a similar methodology.

In the two-dimensional reconstructions obtained by μ-CT, the BC and BCP groups at 14 days presented a hypodense cavity corresponding to the space filled by granulation tissue and osteoid matrix, confirmed in the histological sections. In the 42-day period, there was an increase in the hyperdensity of these neoformed areas, related to mineralized bony trabeculae, limited to the borders of the wound, with some islets more evident in the biostimulated BCP animals. These results are in agreement with the findings of Wang et al. [61], which state that laser photobiomodulation therapy contributes to the physiological process of bone formation in calvarial defects of critically sized, blood-clotted rats.

In the BT and BTP groups, all animals had xenograft particles at the insertion site for up to 42 days, without significant variation in the percentage of the total volume of the biomaterial. This finding can be explained by studies by Galindo-Moreno et al. [62], which related the slow reabsorption of bovine hydroxyapatite with its high porosity and the wide surface area. In other studies, they have demonstrated that plasma membrane receptors in osteoclasts recognize elevated calcium levels around the graft particles, leading to inhibition of the activity of this cell and its evasion [63,64,65].

Through the coronal and transaxial sections, it was possible to observe the correct positioning of the graft in the surgical area, without extravasation of the particles. This is due to the agglutinating action of the fibrin sealant on the graft particles, which ensured a proper fixation. The same findings were observed by Chen et al. [66], Brown, Barker [67], and Scognamiglio et al. [68], who concluded that agglutinating action is a determinant factor to promote graft stability to the recipient bed, avoiding micromotion that could interfere in the biological processes involved in the bone consolidation.

Histologically, at 14 days, the BC and BCP experimental groups had a large amount of loose connective tissue and bone formation limited to the periphery of the defect. These results corroborate studies that affirmed that new bone formation begins at the defect margins, overlapping the dura mater, probably stimulated by growth factors such as morphogenetic proteins, released in this region because of vascular rupture resulting from the surgical procedure and the underlying presence of periosteum, which is a source of mesenchymal stem cells [69].

At study completion, new bone formation remained on the wound margin, with thicker, compact bone trabeculae, and complete closure of the defect occurred by fibrous connective tissue. This cellular behavior agrees with studies that reported as a critical-size bone defect, since the lack of growth and nutrition factors at the center of the surgical area limits osteoblastic differentiation, facilitating the growth of fibroblasts [28,70]. In the same period, the BT and BTP groups presented inflammatory cells involving the xenograft particles, a cellular mechanism inherent to the implantation of biomaterials in tissues in vivo [71,72]. However, this biological response did not elicit an intense inflammatory reaction that could compromise the entire surgical procedure. This can be explained by the nature of materials used in this study, which provide a controlled and bioinert tissue response in the physiological microenvironment [73].

At 42 days, this inflammatory infiltrate showed to be in the resolution phase, being more evident in the group treated with laser photobiomodulation, BTP, a result also observed by Kazancioglu et al. [74] and de Oliveira et al. [75]. Studies have demonstrated the effectiveness of laser photobiomodulation therapy in the inflammatory process by reducing the synthesis of reactive oxygen and nitrogen species (ROS and RNS), leading to modifications in the expression of anti-inflammatory and pro-inflammatory chemical mediator genes [76,77].

Regarding the material volume of the demineralized bovine bone (B), in the BT group there was a slight decrease in the means when compared to BTP (36.20 ± 5.71% to 35.99 ± 7.00%; 37.52 ± 5.75% to 33.85 ± 4.50%, respectively). This may be related to the direct action of laser photobiomodulation in the activation of nuclear factor-κB ligand (RANKL), influencing the maturation and differentiation process of osteoclasts responsible for the degradation of particles [78,79]. At 42 days, there was an increase in new bone formation and decrease in soft tissue volume in BCP group. Scientific studies have reported the positive effect of PBMT on the bone consolidation process by stimulating mitochondrial respiration, and increasing ATP synthesis, local blood flow, and collagen production, and consequently inducing proliferation and differentiation of osteoprogenitor cells [80,81].

When we compare the quantitative data of the previous study [28], carried out on slides, with the present study, quantified in microtomography, we can observe different data. For example, at 42 days, with graft and PBMT, the percentage of new bone formed was 10.64 ± 0.97 on slides and 17.37 ± 4.38 on micro-CT. This occurred because of the difficulty of binarization or separation of the biomaterial from the neoformed bone phase, which cannot be performed correctly because of the isoradiographic density of particles of the material and of the parietal bones [69,82].

Regarding the histochemical analysis of collagen fibers by picrosirius red staining, all groups initially analyzed demonstrated the formation of thin and disorganized type III collagen fibers with a gradual increase in birefringence throughout the periods [59]. At 42 days, the BCP and BTP groups presented a better lamellar organization of collagen fibers, with a transition from birefringence to green, indicative of bone maturation. Research papers demonstrate that the monochromaticity of a laser determines the selective absorption by chromophores present in the osteoblastic cells, stimulating the expression of growth factors, including the canonical fibroblast growth factor (FGFs) [83]. These growth factors are responsible for modulating the differentiation of mesenchymal cells by the FGF/fibroblast growth-factor receptor FGFR (transmembrane tyrosine kinase receptors) signaling mechanism, and consequently type I collagen synthesis, with thicker and more organized fibers [84,85].

In the immunohistochemical results, BMP—2/4, OCN, and VEGF immunostaining were significantly higher in BCP and BTP when compared to the BC and BT groups. Such data are indicative of the positive effect of laser biostimulation from the initial stages of bone matrix synthesis (BMP—2/4) to the final stages of bone mineralization and renewal (OCN) [86]. Furthermore, angiogenesis after tissue injury, marked by the expression of VEGF protein, showed in this investigation that the blood supply to combat the local oxygen deficit was moderate in the two periods analyzed for the biostimulated groups [87]. This fact is attributed to pleiotropic substances synthesized by biostimulation that are involved in the transduction of cellular signals, in the regulation of antioxidant enzymes, chemotaxis, angiogenesis, cell differentiation, and in the modulation of the inflammatory process [88].

In the groups treated with biomaterial, a significantly higher number of TRAP-positive cells was observed, possibly because of the reduction in the rate of resorption by osteoclasts during repair. Conflicting data are presented in these groups when combined with the greater amount of new bone, suggesting that accelerated resorption of graft granules before 30 days is not beneficial to bone formation, which was confirmed by the percentage of new bone tissue formed lower in the groups treated with clot at the end of the experiment [89].

The number of osteocytes in the newly formed bone matrix proportionally reduced in all groups evaluated, following the physiological evolution of the newly formed bone tissue (woven to lamellar bone), which suggests a similar bone maturity for the treatments [90]. In summary, the combination of bone substitute materials and photobiomodulation therapy in bone regeneration attracted the attention of several groups of researchers because it is a coadjutant and a promising method to existing treatments.

We can consider as a limitation of this study the absence of comparative tests with autogenous graft, considered the gold standard, but which would increase the number of animals and is already included in many studies published in the scientific world.

## 5. Conclusions

This study aimed to evaluate the effects of photobiomodulation in the repair of critical bone defects in rat calvaria filled with bone substitute and derived from human blood fibrin sealant. In view of the results obtained, it was concluded that the association of two scaffolds, bone substitute and fibrin sealant, to the proposed protocol for photobiomodulation therapy, with the use of a low-level laser, improved the formation of new bone during the restoration of bone gaps, suggesting it is a promising strategy, mainly for the treatment of large defects (critical size defects).

## Figures and Tables

**Table 1:** Mean ± standard deviation of volume and percentage of structures into defect obtained by 3D-morphometry on micro-CT images.

Parameter	Period (Days)	Groups			
		BC	BCP	BT	BTP
Total volume (TV, mm^3^)	14	36.52 ± 3.59 aA	36.22 ± 3.48 aA	114.0 ± 9.20 bA	112.8 ± 11.84 bA
	42	36.60 ± 3.35 aA	35.80 ± 4.38 aA	115.4 ± 13.3 bA	106.7 ± 13.72 bA
Material volume (MV, mm^3^)	14	-	-	41.09 ± 5.90 aA	42.35 ± 8.32 aA
	42	-	-	41.28 ± 7.73 aA	35.97 ± 5.57 aA
Bone volume (BV, mm^3^)	14	2.93 ± 1.43 aA	3.02 ± 0.71 aA	17.76 ± 3.52 bA	19.88 ± 5.47 bA
	42	5.20 ± 1.02 aB	6.16 ± 2.10 aB	15.56 ± 4.68 bA	18.17 ± 3.04 bA
Soft tissue volume (StV, mm^3^)	14	33.59 ± 2.94 aA	33.20 ± 3.19 aA	50.61 ± 5.34 bA	56.88 ± 9.82 bA
	42	31.40 ± 4.00 aA	29.64 ± 3.00 aA	58.51 ± 13.1 bA	52.61 ± 13.5 bA
Material volume (MV/TV, %)	14	-	-	36.20 ± 5.71 aA	37.52 ± 5.75 aA
	42	-	-	35.99 ± 7.00 aA	33.85 ± 4.50 aA
Bone volume (BV/TV, %)	14	7.89 ± 3.54 aA	8.32 ± 1.60 aA	15.53 ± 2.38 bA	17.56 ± 4.04 bA
	42	14.42 ± 3.66 aB	16.96 ± 4.38 aB	13.44 ± 3.88 aA	17.37 ± 4.38 aA
Soft tissue volume (StV/TV, %)	14	92.10 ± 3.54 aA	91.68 ± 1.60 aA	48.27 ± 7.21 bA	44.92 ± 3.02 bA
	42	85.58 ± 3.66 aB	83.04 ± 4.38 aB	50.57 ± 9.74 bA	48.78 ± 8.16 bA

Different small letters (line comparison, BC vs. BCP vs. BT vs. BTP in each period, 14 or 42 days, a ≠ b) indicate a statistically significant difference. Different capital letters (comparison in columns, 14 vs. 42 days, in each parameter, A ≠ B) indicate a significant difference. Values: mean ± standard deviation. ANOVA followed by Tukey test and Student’s test respectively, are both at *p* < 0.05.

**Table 2:** Mean ± standard deviation of numbers of osteocytes.

Cell Numbers/mm^2^ Bone Matrix	Period (Days)	Groups			
		BC	BCP	BT	BTP
Osteocytes	14	787 ± 111.6 aA	764 ± 222.7 aA	789 ± 80.2 aA	740 ± 43.8 aA
	42	558 ± 50.2 aB	476 ± 87.3 aB	453 ± 65.1 aB	493 ± 79.0 aB

Same small letters (line comparison, BC vs. BCP vs. BT vs. BTP in each period, 14 or 42 days, a = a) indicate that there was no significant difference. Different capital letters (comparison in columns, 14 vs. 42 days, A ≠ B) indicate a statistically significant difference. Values: mean ± standard deviation. ANOVA followed by the Tukey test and Student’s test, respectively, are both at *p* < 0.05.

**Table 3:** Comparison of the scores attributed to the immunostaining for BMP, VEGF, and OCN proteins in the defects of each experimental group by the Kruskal–Wallis test.

Parameter	Period (Days)	Groups: Median (Min–Max)			
		BC	BCP	BT	BTP
BMP	14 days	1(1-1) A	2(2-3) B	1(1-1) A	2(2-2) B
	42 days	1(1-1) A	2(2-3) B	1(1-1) A	2(2-3) B
VEGF	14 days	1(1-1) A	3(2-3) B	1(1-2) A	2(2-2) AB
	42 days	1(1-2) A,B	3(2-3) B	1(1-1) A	3(2-3) B
OCN	14 days	1(1-1) A	2(2-2) B	1(1-1) A	2(1-2) AB
	42 days	1(1-1) A	2(2-2) B	1(1-1) A	2(2-2) B

Different capital letters (comparison in lines, BC vs. BCP vs. BT vs. BTP in each period, 14 or 42 days A ≠ B) indicate a statistically significant difference (*p* < 0.05).

**Table 4:** Mean ± standard deviation of TRAP positive cells.

Cell Numbers/mm^2^ Bone Matrix	Period (Days)	Groups			
		BC	BCP	BT	BTP
TRAP+	14	2.8 ± 0.84 aA	4.8 ± 0.83 bA	20.2 ± 4.02 cA	30.4 ± 1.34 dA
	42	4.0 ± 1.23 aA	5.8 ± 2.19 bA	22.0 ± 2.45 cA	36.6 ± 4.51 dB

Different small letters (line comparison, BC vs. BCP vs. BT vs. BTP in each period, 14 or 42 days, a ≠ b ≠ c ≠ d) indicate a significant difference. Different capital letters (comparison in columns, 14 vs. 42 days, A ≠ B) indicate a significant difference. Values: mean ± standard deviation. ANOVA followed by the Tukey test and Student’s test, respectively, are both at *p* < 0.05.

**Table 5:** Data on materials used in transplant therapy and the corresponding mechanisms of action leading to bone regeneration.

Biomaterials	Characteristics	Mechanism of Action
Bio-Oss^TM^	HAP(h) ∼ HAP (x)	Slow macrophage biodegradability—longer cell support time, Mechanical resistance [47,48],
	70–75% porosity	Extensive surface area > cell adhesion [49],
	Interconnected micro and macropores (300–1500 µm)	Prevents soft tissue invagination into the defect, Osteoconductivity—cell mechanical support [50],
	Deproteinization (≤300 °C)—no organic components	Biocompatibility—non-immunogenic [51],
Tisseel Lyo™	Cross-linked fibrin	Viscoelasticity property—tissue flap stability [52],
	Fibrin polymerization—fibrin polymer	Binding effect of particulate biomaterial [52],
	Aprotinin component (antifibrinolytic)	Cellular support throughout the trial period (∼ 40 days) [53],
	Blood components	Bioactive 3D matrix—binding sites: platelets, endothelial cells, fibroblasts, neutrophils, and macrophages and also for molecules, proteins and growth factors [16], Biocompatibility and biodegradability (fibrinolysis) [54],
	Hemostatic mechanisms	Thrombus—angiogenic cells (surgical hemostasis) [55].

HAP (h)—human hydroxyapatite; HAP (x)—xenogenic hydroxyapatite.

## Data Availability

The data presented in this study are available on request from the corresponding authors. The data are not publicly available because they are part of a doctoral thesis not yet deposited in a public repository. Samples of the Bio-Oss^TM^ matrix are available from the authors.

## Supplementary Materials

The following supporting information can be downloaded at https://www.mdpi.com/article/10.3390/polym14194170/s1: Figure S1: Protocol of the Photobiomodulation. Figure S2: CTan software showing a two-dimensional transaxial image of one of the defects filled by biomaterials and subsequent binarization for the quantification of the biomaterial (A) and the newly formed bone tissue (B) and their respective quantitative results determined by the software (A ‘and B’); Figure S3: Illustration of the morphometric analysis of images stained with picrosirius red to quantify the birefringence of collagen fibers of the newly formed bone by the Axiovision software; Figure S4: Illustration of the quantitative analysis of osteocytes in newly formed bone tissue by the Axio vision software.
