# Supplementary figures and images for: Use of Photobiomodulation Combined with Fibrin Sealant and Bone Substitute Improving the Bone Repair of Critical Defects
Source: Polymers (Basel). 2022 Oct 4;14(19):4170. doi: 10.3390/polym14194170 (PMC9572221; doi:10.3390/polym14194170)

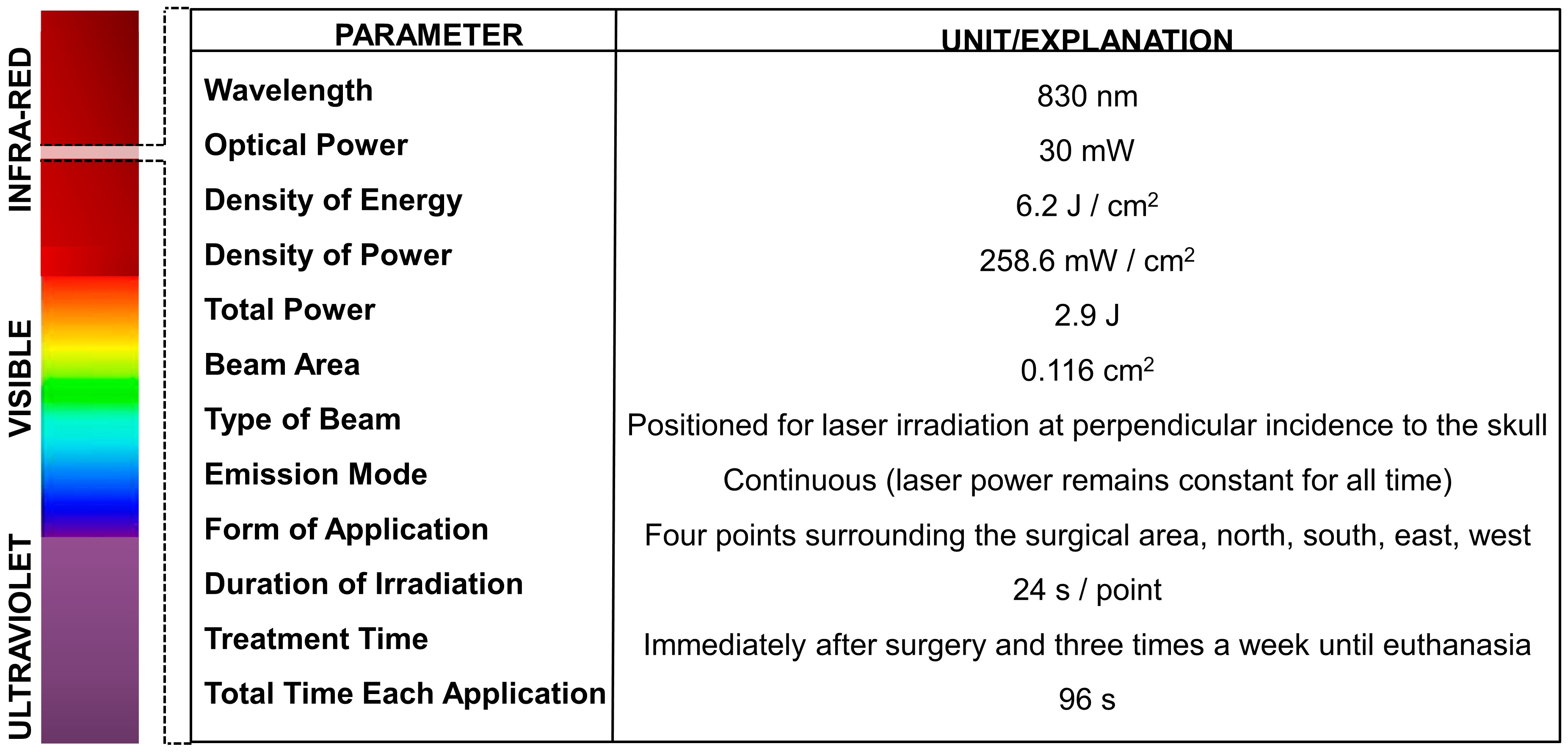

Supplement: Supplementary file 1 [file polymers-14-04170-s001.zip › Figure S1.jpg]

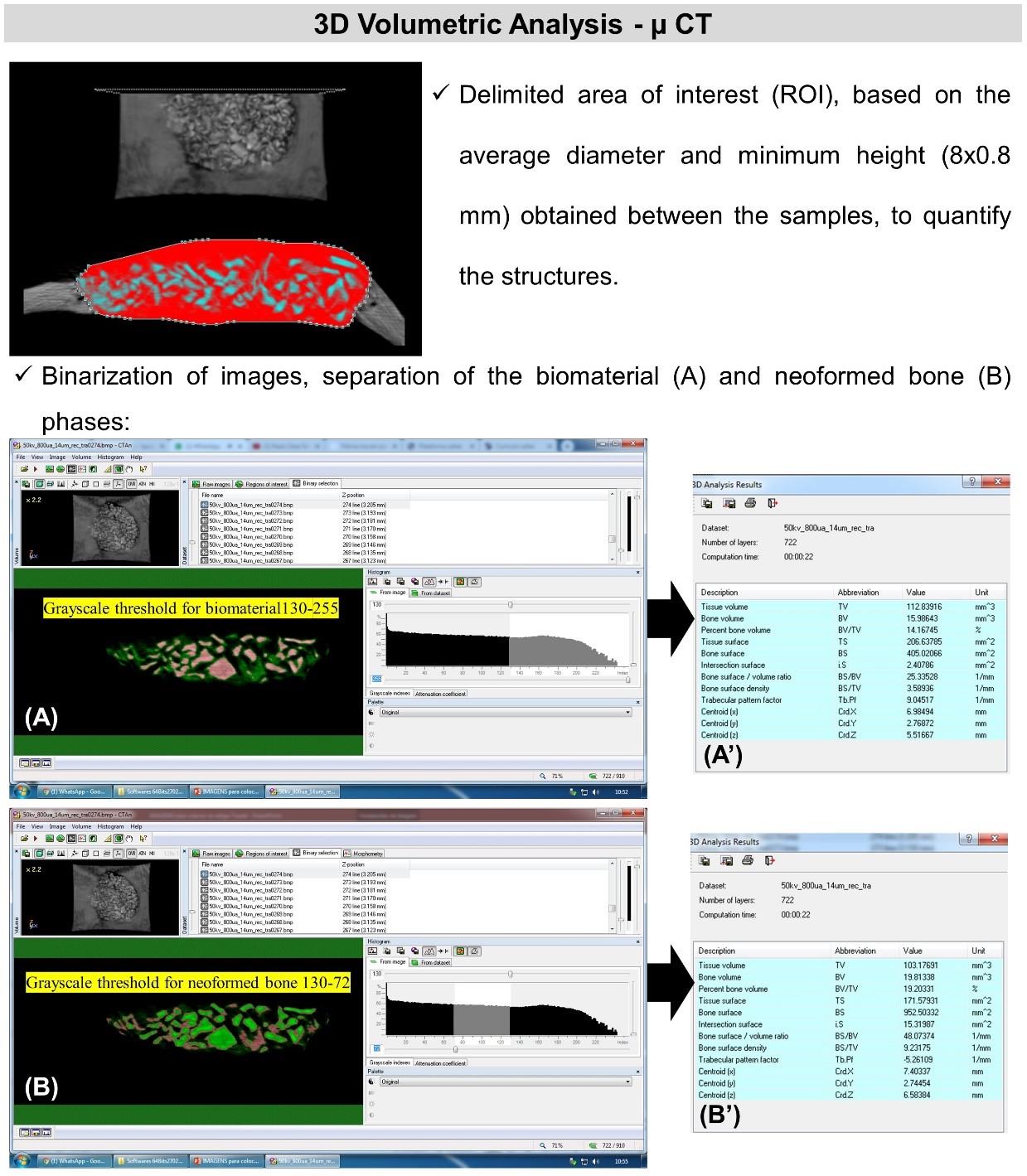

Supplement: Supplementary file 1 [file polymers-14-04170-s001.zip › Figure S2.jpg]

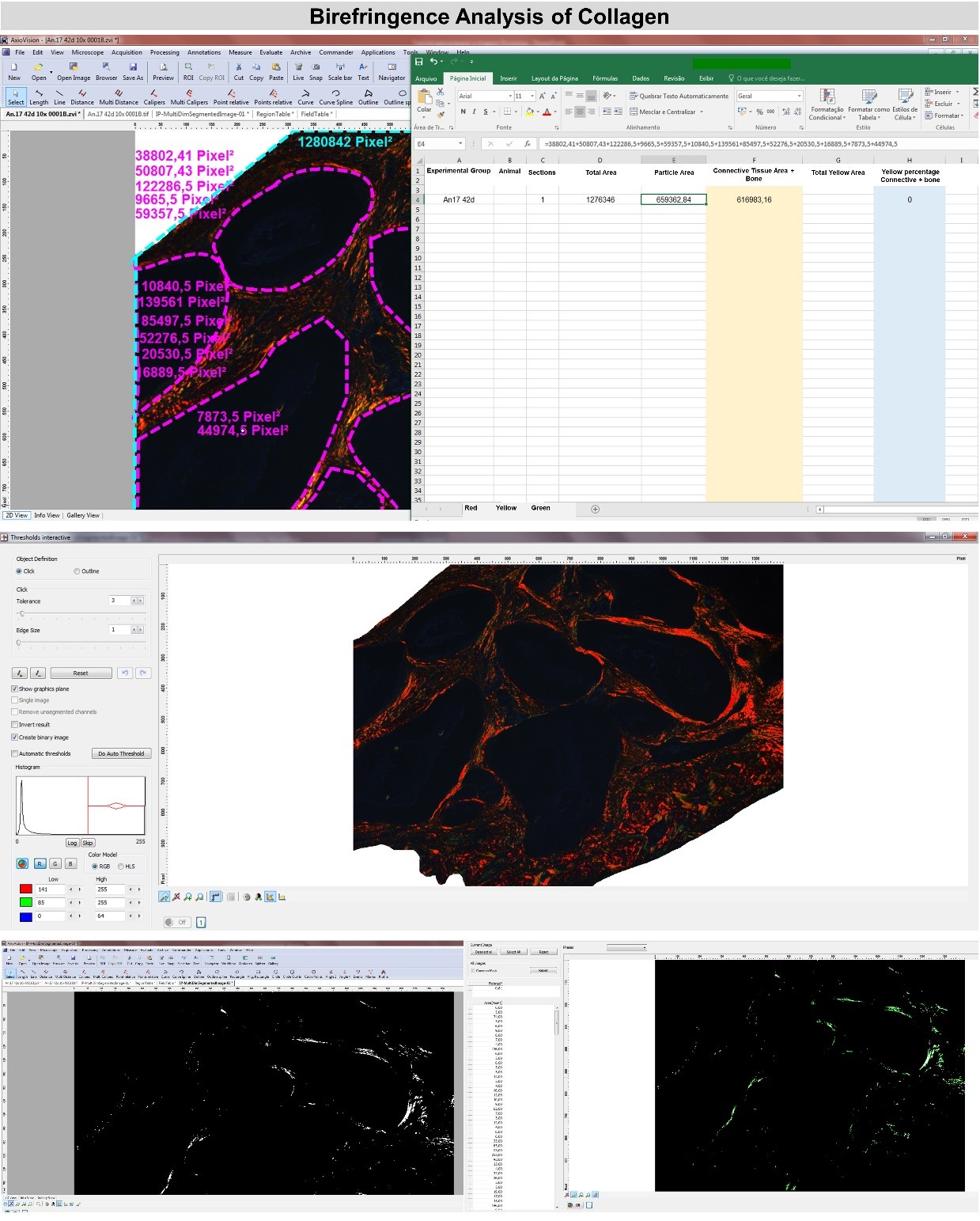

Supplement: Supplementary file 1 [file polymers-14-04170-s001.zip › Figure S3.jpg]

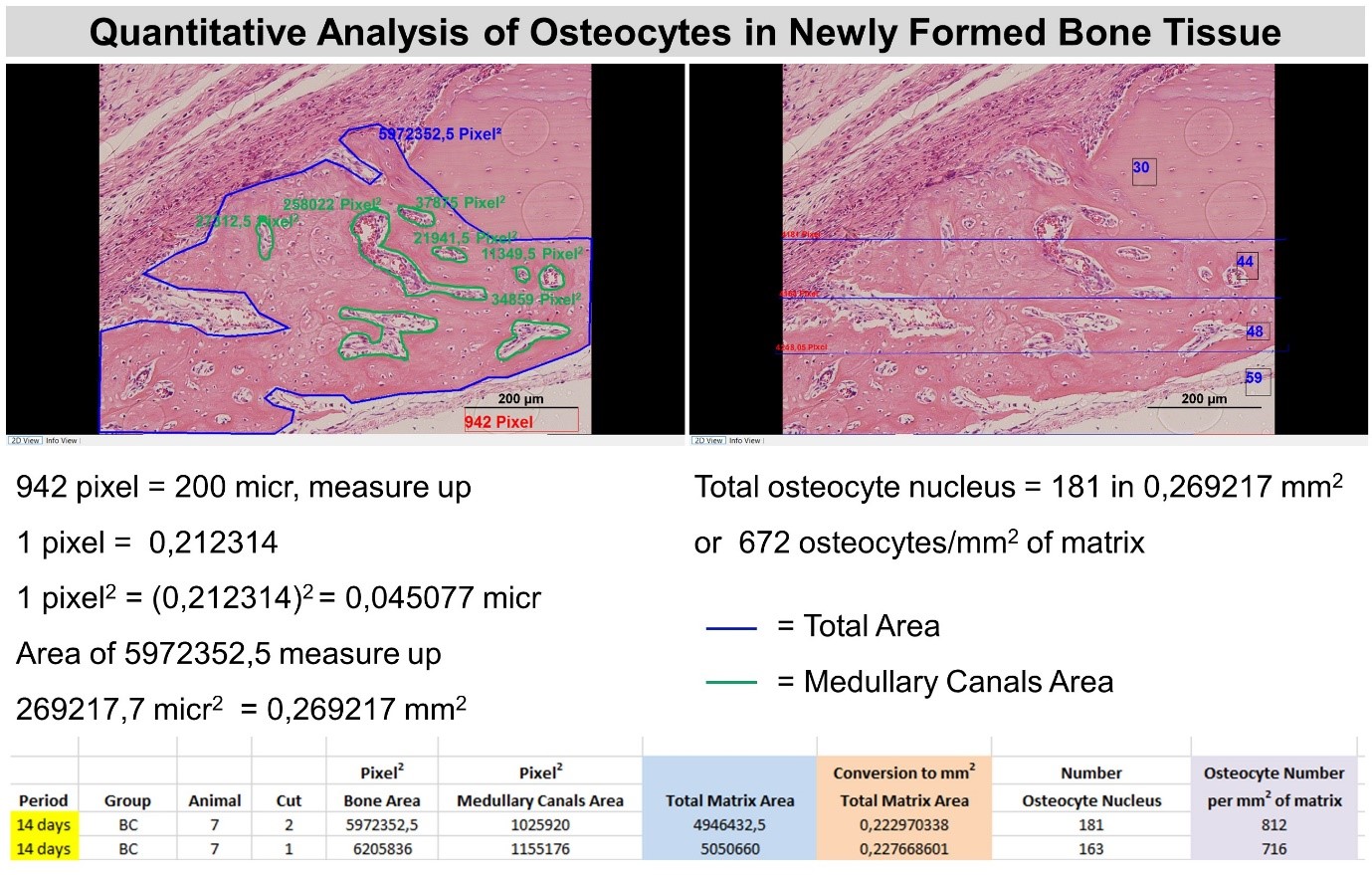

Supplement: Supplementary file 1 [file polymers-14-04170-s001.zip › Figure S4.jpg]
